# Impact of light on *Hypocrea jecorina *and the multiple cellular roles of ENVOY in this process

**DOI:** 10.1186/1471-2164-8-449

**Published:** 2007-12-04

**Authors:** Andrè Schuster, Christian P Kubicek, Martina A Friedl, Irina S Druzhinina, Monika Schmoll

**Affiliations:** 1 Division of Gene Technology and Applied Biochemistry, Institute for Chemical Engineering, Vienna University of Technology, Getreidemarkt 9/1665, A-1060 Wien, Austria.

## Abstract

**Background:**

In fungi, light is primarily known to influence general morphogenesis and both sexual and asexual sporulation. In order to expand the knowledge on the effect of light in fungi and to determine the role of the light regulatory protein ENVOY in the implementation of this effect, we performed a global screen for genes, which are specifically effected by light in the fungus *Hypocrea jecorina *(anamorph *Trichoderma reesei*) using Rapid Subtraction Hybridization (RaSH). Based on these data, we analyzed whether these genes are influenced by ENVOY and if overexpression of ENVOY in darkness would be sufficient to execute its function.

**Results:**

The cellular functions of the detected light responsive genes comprised a variety of roles in transcription, translation, signal transduction, metabolism, and transport. Their response to light with respect to the involvement of ENVOY could be classified as follows: (i) ENVOY-mediated upregulation by light; (ii) ENVOY-independent upregulation by light; (iii) ENVOY-antagonized upregulation by light; ENVOY-dependent repression by light; (iv) ENVOY-independent repression by light; and (v) both positive and negative regulation by ENVOY of genes not responsive to light in the wild-type. ENVOY was found to be crucial for normal growth in light on various carbon sources and is not able to execute its regulatory function if overexpressed in the darkness.

**Conclusion:**

The different responses indicate that light impacts fungi like *H. jecorina *at several cellular processes, and that it has both positive and negative effects. The data also emphasize that ENVOY has an apparently more widespread cellular role in this process than only in modulating the response to light.

## Background

Light is a fundamental abiotic factor and therefore represents a central environmental signal which influences not only phototrophic but in fact rather the majority of living organisms. Light is thereby sensed by chromophore-binding proteins that act as photoreceptors, which transduce the signal to the expression of the genes involved in the respective response [1,2]. In mitosporic fungi, light is primarily known to stimulate morphogenetic functions and processes of reproduction such as phototropism, spore discharge, the development of sexual and asexual structures [3,4], as well as pigmentation which protects against the deleterious effects of UV-light [5,6]. The molecular responses and mechanisms of adaptation to light, especially with respect to circadian rhythmicity are best documented in *Neurospora crassa *[7-9]. In this fungus, all light-induced phenotypes are dependent on at least one of the two regulators *white-collar-1 *(WC-1; [10]) or *white-collar-2 *(WC-2; [11]). These two genes encode proteins, which contain a zinc finger domain and a PAS-domain through which they interact physically to form the "white collar complex [12]." The WC-1 protein also functions as a blue light receptor via its LOV domain and by its binding of an FAD flavin chromophore [13]. Idnurm and Heitman [14] have recently demonstrated that orthologues of the WC-1/WC-2 proteins of *N. crassa *are present in ascomycetes and basidiomycetes, and thus represent an evolutionary ancient conserved system for the control of light-dependent processes.

The light perception system of *N. crassa *also comprises the small PAS/LOV domain protein VIVID which is believed to act as a modulator of the light response in *N. crassa*. [15-17]. It is a member of the LOV-domain subfamily of PER, ARNT and SIM (PAS)-domain proteins which mediate both ligand binding and protein-protein interactions [18]. VIVID is capable of binding a flavin chromophore [17,19,20]. It has been shown to be localized in the cytoplasm and influences the transient phosphorylation of WC-1 [15,16,21]. The predominant influence of VVD is on the speed with which a transcriptional response to light decays. A loss of VVD causes the clock to be more responsive to light and consequently, circadian gating – the action of the clock to reduce the responses at certain times of day – is muted without VVD [15].

While orthologues of WC-1 and WC-2 have been identified and characterized from various fungi [14], information about possible orthologues of VIVID in other organisms is scarce. Only its orthologue in the ascomycete *Hypocrea jecorina*, Envoy – which has a high similarity to VIVID but is unable to replace it – has recently been characterized [22]. Comparably to *N. crassa vvd, env1 *shows a fast and strong transcriptional response to illumination on several carbon sources.

*H. jecorina *is well known to science because of the use of its anamorph *Trichoderma reesei *as an industrial producer of cellulases and hemicellulases [23-25]. The expression of its cellulase genes depends on the presence of an inducer such as cellulose, lactose, sophorose or L-sorbose, but is otherwise independent of most other nutrients except for a susceptibility of some – but not all of its – genes to partial carbon catabolite repression [26]. Interestingly, however, light stimulates cellulase gene expression in *H. jecorina*, and this stimulation is regulated by ENVOY: a mutant lacking the PAS-domain of ENVOY (*env1*) exhibits an altered cellulase gene transcription pattern both in the presence and absence of light, thus showing that *env1 *is directly or indirectly impacting *cbh1 *gene expression in the darkness [22]. In addition, the loss of the PAS-domain of ENVOY led to an altered transcriptional response of the truncated transcript of *env1*, thus suggesting a regulatory feedback being operative.

Our detection of a light-dependence of cellulase gene transcription of the PAS/LOV domain protein ENVOY raised the question whether there would be more cellular functions in fungi which are controlled by either light and/or ENVOY. To address this question we have performed a genome wide screening for genes regulated by the presence of light, using Rapid Subtraction Hybridization (RaSH). Genes thereby identified were investigated for whether their response would be dependent on a functional *env1 *gene. We will show that light affects transcription of genes of *H. jecorina *both positively as well as negatively, and that for both effects *env1*-independent variants are found. In addition, we will show that ENVOY also acts as a light-independent repressor for several genes, is crucial for normal growth in light on several carbon sources, but is not able to fully execute its regulatory function when overexpressed in darkness. Our data suggest a function of ENVOY in coordination of the light signal with other environmental signals, which is comparable to the gating function shown for VVD of *Neurospora crassa*.

## Results

### Isolation of expressed sequence tags which are differentially expressed in *H. jecorina *after transfer from dark to illumination by light

We used mRNAs from *H. jecorina *QM 9414 pregrown in the dark, and mRNAs from the same strain after subjection to illumination to screen for mRNAs which are more abundant under the latter conditions and thus upregulated by light applying Rapid Subtraction Hybridization [27]. To prevent missing transcripts with only transient accumulation or slower response upon receipt of the light pulse, mRNAs were isolated from mycelia after 15 and 30 min of incubation in light, and combined. As we applied a relatively low stringency, we expected to be able to detect not only genes which are absent during darkness but generally such genes which exhibit a different abundance under these both conditions.

Consequently, 300 putatively positive ESTs were isolated and tested by Reverse Northern blotting [27-29]. The lengths of the respective cDNA sequences were 150 – 400 bp on an average. Based on the signal intensity on the Reverse Northern Blot, 154 EST fragments which were clearly differentially expressed between darkness and illumination and which represented 24 different genes were consecutively chosen for further investigation. As an influence of light on signaling processes can be assumed, additionally several genes involved in signal transduction processes or response to stress were included in the analysis (Table 1).

**Table 1:** Additional genes added to the analysis

Gene(s)	Encoded protein	Function
tre34179 and tre37417	S-adenosyl methionine dependent methyl transferase	Increased methylation of DNA in response to stress leads to decreased transcription [69];
*tmk3*	MAPkinase	Involved in signal transduction; yeast homologue HOG1 regulates glycogen phosphorylase [70]. Glycogen content of *H. jecorina *decreases upon illumination [71]
*hac1*	Transcription factor	Transcription factor involved in regulation of unfolded protein response [34]
*thi4*	Thiazole biosynthetic enzyme	Involved in the biosynthesis of thiazols and in DNA damage response, Fusarium homologue is induced under stress conditions [32]
*gph1*	Glycogen phosphorylase	Involved in degradation of glycogen; glycogen content is decreased upon illumination in *H. jecorina *[71]
*cpc1*	Transcription factor	Cross pathway control protein 1; component of the cross pathway control machinery, involved in activation of amino acid biosynthesis, induced under secretion stress [55]

Since the Reverse Northern blot provides only preliminary information (e.g. some plasmids could contain more than one insert etc.), we assessed the response of expression of all genes to light by Northern blotting. This investigation proved that among the genes analyzed, 20 were indeed significantly (> 40% change in signal intensity) upregulated upon illumination. Interestingly, four genes were shown to be actually repressed by light, one of them representing a false positive result of RaSH regarding the aim of the assay, while the others were three of the genes which – because of their roles in signaling – were intentionally included in the analysis. Since we will present these data in a broader context below, they are not given at this place. For three genes neither significant light- nor ENVOY dependent regulation was detected and therefore they are not discussed further. We also noted that the fluctuations in transcript abundance, which were also seen earlier with the light regulatory gene *env1 *upon cultivation after onset of illumination in minimal medium with 1% glycerol as carbon source [22] or *N. crassa ccg-2 *and NC2B7 (see Figure 4A in [8]) also occur for many of the genes investigated here.

### Gene identification

In order to identify the genes corresponding to the ESTs isolated, we used them to BLAST the *Trichoderma reesei *genome database v2.0 and retrieved the corresponding full length proteins and the correspondingly annotated gene models. The protein sequences were checked for conserved domains using the NCBI CDD search to allow for assigning a function to the respective gene (Table 2). In all cases, the proteins were also blasted against the NCBI database in order to identify the nearest neighbour of the respective gene in *Gibberella zeae *(*Fusarium graminearum*), *Neurospora crassa *and *Saccharomyces cerevisiae *(Table 3). Four of the 24 genes encoded hypothetical proteins, which were also conserved in other fungi, but for which no function could be assigned. Two genes (*env1 *and *phr1*) encoded ENVOY and photolyase 1, respectively, which have already been reported to be up-regulated by light [22,30] and thus confirm the validity of our approach. A high number of genes encoded proteins involved in energy metabolism (i.e. NAD synthase tre9347, succinate dehydrogenase tre20863, a major facilitator sugar transporter tre39397, IMP dehydrogenase tre42719) and protein synthesis (i.e. ribosomal protein L7 *rpl7*), indicating that illumination results in increased respiratory energy production. Other genes isolated are involved in stress response, such as a toxin efflux pump of the major facilitator superfamily (tre10571), genes involved in protein degradation (polyubiquitin *ubi4*, 4-hydroxibenzoate-polyprenyltransferase tre16112, dipeptidyl peptidase III tre39031), nucleotide degradation (IMP pyrophosphatase tre22454) and the thiazole biosynthetic enzyme *thi4*, the homologue of which is upregulated under stress in *Fusarium oxysporum *[31,32]. Finally, genes related to early steps in signal transduction (the mitogen activated protein kinase *tmk3*, the cross pathway control protein *cpc2*) were also upregulated by light. The remaining genes encoded proteins of unclear function in the physiology of *H. jecorina*, such as a predicted porphyromonas-type peptidyl-arginine deimidase (tre45629) which causes citrulinylation of proteins, and a putative CAP20 virulence related protein tre41865, an orthologue of which is involved in appressorium formation and virulence in *Colletotrichum gloeosporioides *[33].

**Table 2:** Protein domains of genes identified by RaSH

***Gene***	**Amino acids**	**Protein domain**	**E-value**	**% aligned**	**Position**
***cpc2***	292	WD40	2.0E-60	93.1%	3–286
***phr1***	591	Deoxyribodipyrimidine photolyase	7.0E-124	99.3%	96–584
		FAD_binding_7	2.0E-97	100%	316–587
***rpl7***	248	Ribosomal_L7	5.0E-66	100%	88–247
		Ribosomal_L30	2.0E-14	100%	87–139
***ubi4***	305	Ubiquitin	7.0E-33	100%	1–76
		Ubiquitin	7.0E-33	100%	77–152
		Ubiquitin	7.0E-33	100%	153–228
		Ubiquitin	7.0E-33	100%	229–304
***tre9347***	700	NAD_synthase	3.0E-59	99.2%	328–653
		Carbon-nitrogen hydrolase	1.0E-18	100%	6–201
		Predicted amidohydrolase	4.0E-27	91.6%	5–282
***tre10571***	534	Major facilitator superfamily MFS_1	4.0E-12	100%	31–443
		Arabinose efflux permease	7.0E-13	46.7%	26–208
		Fungal trichothecene efflux pump (TRI12)	2.0E-06	34.3%	102–303
***tre16112***	304	Hydroxybenzoate polyprenyltransferase	2.0E-32	99.7%	2–289
		UbiA prenyltransferase family	5.0E-23	100%	24–301
***tre20863***	648	Succinate dehydrogenase/fumarate reductase	2.0E-168	100%	56–631
		FAD binding domain	2.0E-140	99.4%	162–492
		Aspartate oxidase	1.0E-92	93.8%	85–618
		Fumarate reductase/succinate dehydrogenase flavoprotein C-terminal domain	4.0E-44	100%	513–648
***tre22454***	180	NTPase/HAM1	1.0E-54	100%	5–178
		Xanthosine triphosphate pyrophosphatase	2.0E-45	96.9%	5–180
***tre22667***	193	Ribosomal protein L6P/L9E	4.0E-30	97.2%	1–187
		Ribosomal protein L6	7.0E-07	100%	97–180
***tre31929***	270	Adenylate kinase	3.0E-75	100%	44–231
		Adenylate kinase, active site lid	2.0E-12	100%	167–202
***tre35050***	104	-	-	-	-
***tre39031***	711	Peptidase_M49	6.0E-170	98.8%	143–709
***tre39397***	465	Sugar (and other) transporter	7.0E-13	87.1%	44–462
***tre40105***	331	-	-	-	-
***tre41865***	183	Perilipin	2.0E-04	27.4%	15–110
***tre42719***	537	IMP dehydrogenase/GMP reductase domain	1.0E-159	99.4%	42–526
		CBS domain	3.0E-13	93.2%	130–236
		Predicted transcriptional regulator	7.0E-09	36.7%	130–238
		NAD(P)H-dependent flavin oxidoreductase (oxidored) FMN-binding superfamily domain	1.0E-04	99.1%	195–397
***tre45629***	356	Porphyromonas-type peptidyl-arginine deiminase	1.0E-67	100%	6–353
***tre72859***	114	-	-	-	-

**Table 3:** Blast analysis of genes identified by RaSH

**Gene**	**Best Hit**	**E-Value**	***Fusarium spp*.**	**E-Value**	***Neurospora crassa***	**E-Value**	***Saccharomyces cerevisiae***	**E-Value**
***cpc2***	XP_390046.1 Guanine nucleotide-binding protein beta subunit [*G. zeae*]	2.00E-169	XP_390046.1 Guanine nucleotide-binding protein beta subunit	2.00E-169	Q01369| GBLP_NEUCR WD-repeat protein cpc-2	1.00E-166	NP_013834.1 Asc1p	2.00E-94
***phr1***	CAA08916.1 DNA photolyase [*Hypocrea lixii*]	0.0	XP_380973.1 hypothetical protein FG00797.1	0.0	P27526| PHR_NEUCR Deoxyribodipyrimidine photolyase	0.0	P05066| PHR_YEAST Deoxyribodipyrimidine photo-lyase	2.00E-89
***rpl7***	XP_382718.1 conserved hypothetical protein [*G. zeae*]	1.00E-112	XP_382718.1 conserved hypothetical protein	1.00E-112	XP_962950.1 hypothetical protein	4.00E-108	NP_011439.1 Rpl7ap	2.00E-77
***tre10571***	XP_388925.1 hypothetical protein FG08749.1 [*G. zeae*]	0.0	XP_388925.1 hypothetical protein FG08749.1	0.0	XP_330290.1 hypothetical protein	4.00E-162	NP_011740.1 Azr1p	2.00E-44
***tre16112***	XP_327992.1 h. p. (AL451012) related to para-hypolyprenyltransferase precursor [*N. crassa*]	1.00E-124	XP_390908.1 hypothetical protein FG10732.1	2.00E-110	-	-	NP_014439.1 Coq2p	1.00E-56
***tre20683***	EAQ93406.1 conserved hypothetical protein [*Chaetomium globosum CBS *]	0.0	XP_387537.1 hypothetical protein FG07361.1	0.0	XP_965239.1 hypothetical protein	0.0	Q00711| DHSA_YEAST Succinate dehydrogenase	0.0
***tre22454***	XP_955963.1 hypothetical protein [*N. crassa N150*]	7.00E-76	XP_387647.1 hypothetical protein FG07471.1	4.00E-73	XP_955963.1 hypothetical protein [Neurospora crassa N150]	7.00E-76	NP_012603.1 Ham1p	2.00E-33
***tre22667***	XP_381330.1 hypothetical protein FG01154.1 [*G. zeae*]	2.00E-88	XP_381330.1 hypothetical protein FG01154.1	2.00E-88	XP_965129.1 hypothetical protein	1.00E-87	NP_014332.1 Rpl9bp	2.00E-63
***tre31929***	XP_390913.1 Probable adenylate kinase (ATP-AMP transph [*G. zeae*]	1.00E-121	XP_390913.1 Probable adenylate kinase	1.00E-121	XP_956253.1 probable adenylate kinase [MIPS]	2.00E-116	NP_010512.1 Adk1p	4.00E-89
***tre35050***	XP_001230117.1 hypothetical protein CHGG_03601 [*Chaetomium globosum*]	1.00E-23	XP_386761.1 hypothetical protein FG06585.1	1.00E-15	XP_956091.1 hypothetical protein	7.00E-21	AAB50692.1 Paf1p	1.7
***tre39031***	XP_381193.1 hypothetical protein FG01017.1 [*G. zeae*]	0.0	XP_381193.1 hypothetical protein FG01017.1	0.0	CAE76510.1 probable dipeptidylpeptidase III	0.0	Q08225| DPP3_YEAST Dipeptidyl aminopeptidase III	2.00E-163
***tre39397***	XP_369043.1hypothetical protein MG00201.4 [*Magnaporthe grisea*]	0.0	XP_388057.1 hypothetical protein FG07881.1	0.0	XP_326778.1 hypothetical protein	1.00E-86	-	-
***tre40105***	BAE58733.1 unnamed protein product [*Aspergillus oryzae*]	3.00E-54	XP_384339.1 hypothetical protein FG04163.1	8.00E-38	XP_960170.1 hypothetical protein	7.00E-26	-	-
***tre41025***	EAS32414.1 predicted protein [*Coccidioides immitis RS*]	2.00E-21	XP_384237.1 hypothetical protein FG04061.1	5.00E-16	XP_959109.1 hypothetical protein	0.001	NP_012284.1 Muc1p	0.038
***tre41865***	XP_385353.1hypothetical protein FG05177.1 [*G. zeae*]	2.00E-62	XP_385353.1hypothetical protein FG05177.1	2.00E-62	CAD70317.1 probable CAP20-virulence factor	4.00E-35	-	-
***tre42719***	XP_964976.1 hypothetical protein [*N. crassa*]	0.0	XP_381037.1 conserved hypothetical protein	0.0	XP_964976.1 hypothetical protein	0.0	NP_013656.1 Imd4p	0.0
***tre45629***	XP_748505.1 Porphyromonas-type peptidyl-arginine deiminase superfamily [*Aspergillus fumigatus Af293*]	2.00E-62	-	-	-	-	-	-
***tre72859***	XP_381443.1 hypothetical protein FG01267.1 [*G. zeae*]	7.00E-28	XP_381443.1 hypothetical protein FG01267.1	7.00E-28	XP_964260.1 hypothetical protein	1.00E-23	Q07953| YL022_YEAST UPF0023 protein YLR022C	0.014
***tre9347***	XP_387574.1 hypothetical protein FG07398.1 [*G. zeae*]	0.0	XP_387574.1 hypothetical protein FG07398.1	0.0	XP_959191.1 hypothetical protein	0.0	NP_011941.1 Qns1p	0.0
***ubi4***	XP_460488.1 protein DEHA0F03157g [*Debaryomyces hansenii CBS767*]	2.00E-168	XP_388944.1 protein FG08768.1	2.00E-124	XP_958803.1 polyubiquitin	2.00E-166	NP_013061.1 Ubi4p	9.00E-168

### Light-dependent upregulation of gene expression can occur in *env1*-dependent, *env1*-independent, and *env1*-antagonized ways

**Figure 1:**
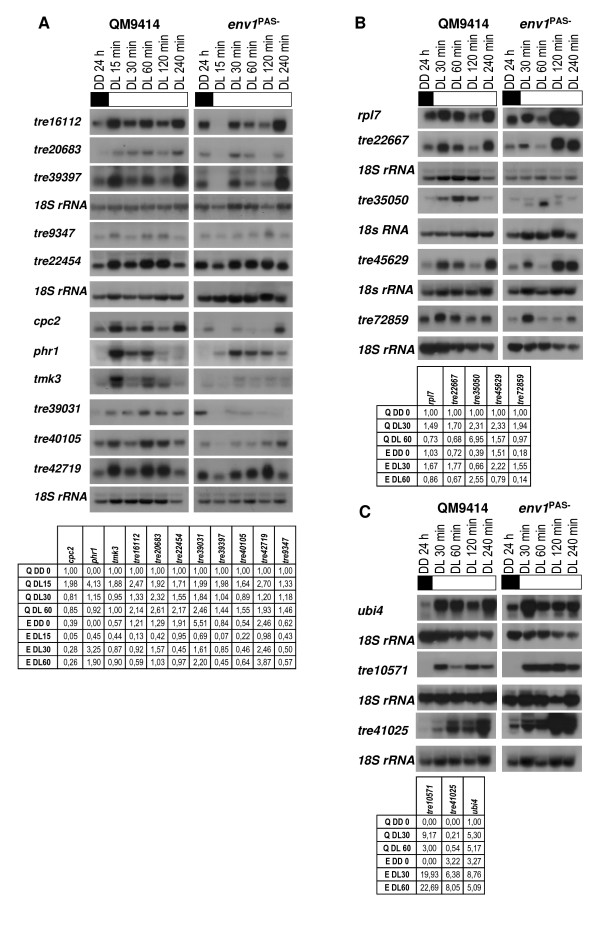
**Northern analysis of light- and *env1*-responsive genes**. Strains were grown on Mandels Andreotti minimal medium with 1% (w/v) glycerol as carbon source for 24 h in darkness (DD) and harvested after the indicated time (DL) of illumination (1800 lux, 25 μmol photons m^-2^s^-1^). A representative hybridization with 18S rRNA for every set of Northerns is given below the respective series. Transcript abundance is given below the blots and was measured for wild-type QM9414 (Q) and *env1*^PAS- ^by densitometry to verify up-regulation until 60 min of illumination, related to 18S rRNA and normalized to the dark control of the wild-type strain (QM9414, 24 h, DD). If no transcript was detected in QM9414 in darkness, the values represent signal strength above background. (A) Transcription of genes upregulated by light but not in the *env1*^PAS- ^strain. (B) Transcription of genes upregulated both by light and in the *env1*^PAS- ^strain. (C) Transcription of genes upregulated by light, which show increased upregulation in the *env1*^PAS- ^strain.

Having identified a reasonable set of genes which were found to be upregulated in *H. jecorina *by light, we now investigated whether they would indeed require the function of *env1 *for this purpose. To this end, we compared the expression profile of these genes in *H. jecorina *QM 9414 to that of the *env1*^PAS- ^strain over a period of 240 min. This strain lacks the PAS-domain of the light regulatory protein ENVOY and shows altered response to light as well as a considerably decreased light tolerance [22]. The corresponding results showed that ENVOY appears to play in fact at least three different roles in light regulation: eleven of the genes (tre16112, tre20683, tre39397, tre9347, tre22454, *cpc2, phr1*, *tmk3*, tre39031, tre40105, and tre42719) showed a behaviour which was consistent with the default expectation: a transient upregulation by light, which was not seen in the *env1*^PAS- ^strain and therefore at least partially regulated *env1 *(Fig. 1A), because a response to light nevertheless occurred indicating further light dependent regulators being operative.

In contrast, five genes (*rpl7*, tre22667, tre35050, tre45629 and tre72859), while also showing this upregulation by light, did so also in the *env1*^PAS- ^mutant. Despite the fact that ENVOY seems to be involved in their regulation due to the altered transcription pattern in *env1*^PAS-^their response to light by increased transcription is not exclusively dependent on ENVOY (Fig. 1B).

In addition, three other genes (*ubi4*, tre10571, tre41025) also exhibited significant upregulation of gene expression upon exposure to light, but this upregulation was even stronger in the *env1*^PAS- ^mutant, indicating that ENVOY antagonizes this activation in the wild-type strain (Fig. 1C). Since this enhanced transcription in the mutant strain also occurs in darkness with *ubi4 *and tre41025, these genes seem to be subject to a general repression by ENVOY.

### Light repression of gene expression can occur in *env1*-dependent and *env1*-independent manners

**Figure 2:**
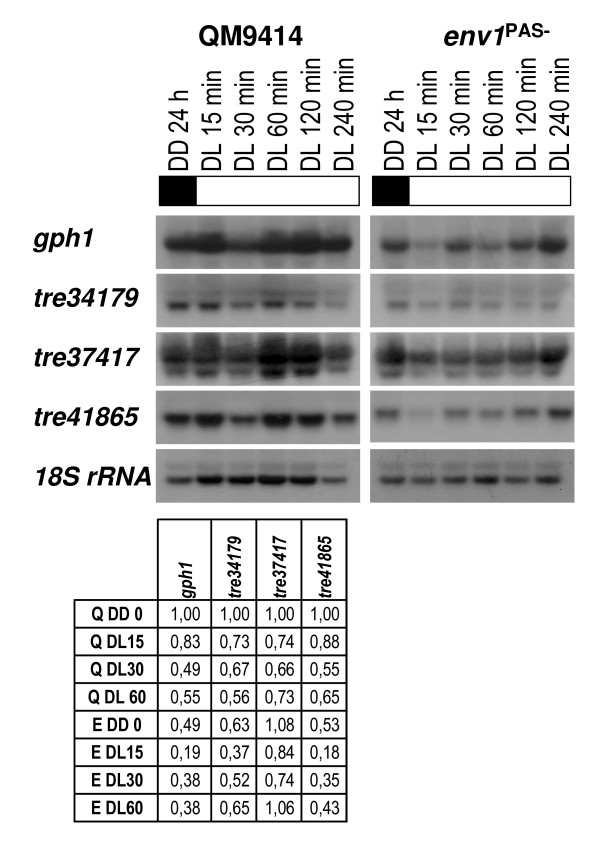
**Northern analysis of genes showing decreased transcription upon illumination**. Strains were grown on Mandels Andreotti minimal medium with 1% (w/v) glycerol as carbon source for 24 h in darkness (DD) and harvested after the indicated time (DL) of illumination (1800 lux, 25 μmol photons m^-2^s^-1^). A representative hybridization with 18S rRNA for every set of Northerns is given below the respective series. Transcript abundance is given below the blots and was measured for wild-type QM9414 (Q) and *env1*^PAS- ^by densitometry to verify up-regulation until 60 min of illumination, related to 18S rRNA and normalized to the dark control of the wild-type strain (QM9414, 24 h, DD). If no transcript was detected in QM9414 in darkness, the values represent signal strength above background.

Four genes were noted, whose mRNA abundance decreased upon exposure to light: *gph1*, tre34179, tre37417 and tre41865. Interestingly, their dependence on *env1 *showed a different influence: expression of tre37417 was not significantly regulated by light in the *env1*^PAS- ^mutant, and that of *gph1*, tre34179 and tre41685, which intrinsically represents a false positive result with respect to the aim of the study, decreased (Fig. 2). These data show that ENVOY can also act as an antagonist of the negative effect of light on gene expression. Moreover, obviously there is – as expected – also an additional, *env1*-independent, pathway of light regulation of gene expression.

### *env1 *also regulates expression of genes which do not respond to light

**Figure 3:**
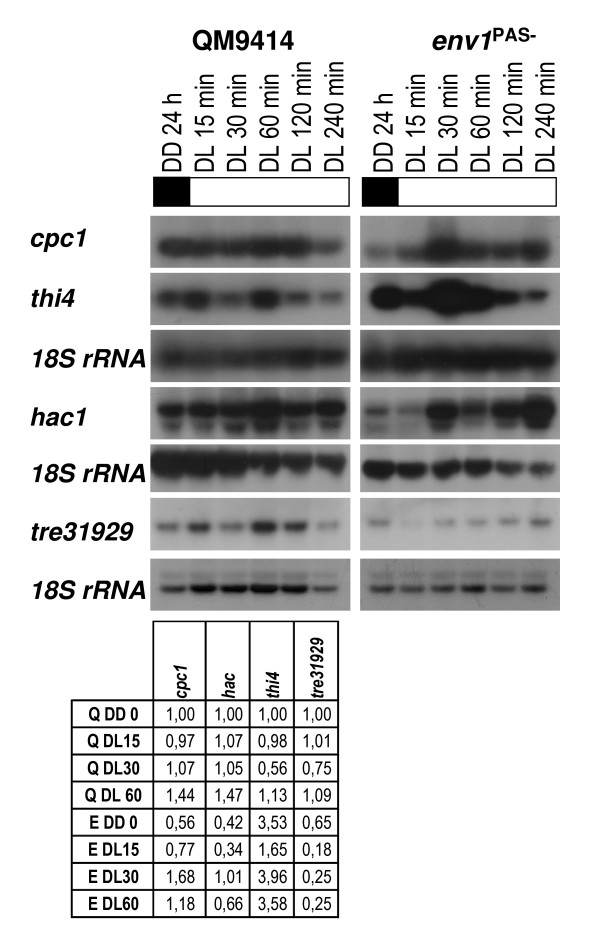
**Northern analysis of genes lacking response to light, but whose transcription is impacted by *env1***. Strains were grown on Mandels Andreotti minimal medium with 1% (w/v) glycerol as carbon source for 24 h in darkness (DD) and harvested after the indicated time (DL) of illumination (1800 lux, 25 μmol photons m^-2^s^-1^). A representative hybridization with 18S rRNA for every set of Northerns is given below the respective series. Transcript abundance is given below the blots and was measured for wild-type QM9414 (Q) and *env1*^PAS- ^by densitometry to verify up-regulation until 60 min of illumination, related to 18S rRNA and normalized to the dark control of the wild-type strain (QM9414, 24 h, DD). If no transcript was detected in QM9414 in darkness, the values represent signal strength above background.

Among the genes analyzed, five genes showed only a minor response (below ± 40% of control) to the presence of light (*cpc1*, *thi4, hac1*, tre31929). Among them *thi4 *was found to be significantly up-regulated in the *env1*^PAS- ^strain indicating repression by ENVOY. The other three exhibited significantly lower transcript abundance in the *env1*^PAS- ^strain and thus are apparently dependent on a function of *env1*, which is not directly related to light response (Fig. 3). Nevertheless, we did not observe an alteration in transcript length of *hac1 *[34] after illumination or due to the lack of a functional ENVOY, what would indicate onset of unfolded protein response due to enhancement of *hac1 *translation after splicing of an intron and alteration of the open reading frame [34].

### Regulatory elements putatively responsible for light response

In order to investigate the significance of certain promoter elements in regulation of light response and as targets of *envoy*-mediated regulation we analyzed 1000 bp of the upstream regions of the genes described in this study (Table 4). Therefore we selected motifs which have been described to play a role in light dependent gene regulation or for which such a function could be expected. EUM1 and EUM2 have been identified in the promoters of the strongly light regulated *env1*-gene and its *N. crassa *orthologue *vvd1*. EUM1 was also found in the *H. jecorina *white collar homologues *blr-1 *and *blr-2 *[22]. The APE-motif (*al-3*-proximal element) is present in the promoter of the *N. crassa *light response output gene *albino-3 *as well as in other light-regulated genes of *Neurospora*. Deletion of this motif abolished the difference in mRNA levels of *al-3 *in light and darkness [35]. The GATA-box is known to be a target of GATA-type zinc finger transcription factors [36] such as the White collar complex (WCC). However, the binding site of this complex shows a variation of the common GATA-consensus in the *N. crassa frq*-promoter and is known as LRE (light response element; [12,37]). The consensus sequence for LRE is GATNC-CGATN, where N can be any nucleotide but the same nucleotide is used in both repeats. The stress element AGGGG is essential for response of *S. cerevisiae *to osmotic stress [38]. However, for *H. atroviridis *this element has been shown not to be sufficient for induction of a certain gene during osmotic stress [39].

**Table 4:** Regulatory motifs within the promoters of the genes analyzed in this study

**Gene**	**EUM1**	**EUM2**	**APE**	**GATA**	**AGGGG**	**LRE**
*cpc1*	0	0	1	2	2	0
*cpc2*	0	0	1	4	0	0
*gph1*	0	0	0	3	6	2
*hac1*	1	0	0	1	7	0
*phr1*	1	0	3	2	1	0
*rpl7*	1	0	0	0	3	0
*thi4*	0	0	0	1	7	0
*tmk3*	2	0	0	2	2	1
*ubi4*	0	0	2	2	5	0
*tre9347*	1	0	0	2	1	0
*tre10571*	0	0	0	0	6	1
*tre16112*	0	0	0	2	0	1
*tre20683*	1	0	2	3	2	0
*tre22454*	0	0	0	1	1	2
*tre22667*	0	0	0	3	1	1
*tre31929*	0	0	0	2	1	0
*tre34179*	1	0	0	5	2	0
*tre35050*	0	0	0	3	0	0
*tre37414*	0	0	0	4	1	2
*tre39031*	1	0	0	1	2	0
*tre39397*	0	1	0	2	0	1
*tre40105*	1	1	0	2	0	0
*tre41025*	2	0	0	1	0	0
*tre41865*	1	0	1	3	3	1
*tre42719*	0	0	0	4	2	1
*tre45629*	1	0	1	5	0	0
*tre72859*	0	0	0	1	2	0

**Figure 4:**
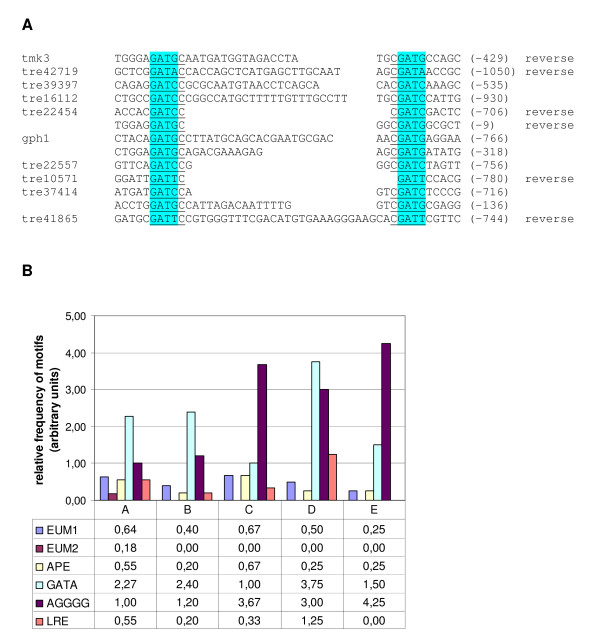
**Analysis of promoter motifs present in light- and/or *env1*-responsive genes**. (A) Alignment of LRE-motifs; the extension of the motifs was limited to 50 bp, only true LRE motifs comprising the GATNC – CGATN consensus with N being the same nucleotide in both repeats were included. (B) Distribution of the respective promoter motifs among the regulatory characteristics of ENVOY as determined by Northern analysis (Figures 1 – 3). The total number of motifs present in one group was related to the number of genes of this group. A: genes upregulated by light but not in the *env1*^PAS- ^strain; B: genes upregulated both by light and in the *env1*^PAS- ^strain; C: genes upregulated by light, which show increased upregulation in the *env1*^PAS- ^strain; D: genes showing decreased transcription upon illumination; E: genes lacking response to light, but whose transcription is impacted by *env1*.

Our analysis revealed motifs which could be responsible for light-dependent regulation in every gene. We grouped the analysis of the promoter motifs according to the suggested function of *env1 *(Figure 4). Interestingly, in the promoters of those genes which are not responsive to light, but regulated by *env1 *the STRE-element AGGGG was overrepresented and the same was the case for those which were upregulated in the *env1*^PAS- ^mutant. This finding may suggest a role of *env1 *in stress response. The phenotype of *env1*^PAS- ^shows a severe perturbation of growth during adaptation to light, which could indicate a role of *env1 *in light-dependent stress management. On the other hand, the GATA-sequence is underrepresented for genes upregulated in the *env1*^PAS- ^mutant. For all other sequence motifs no connection to a specific *env1*-related function or light response in general could be supported. This could at least in part be due to the fact that we cannot distinguish between direct and indirect influences of *env1*, since because of the lack of a known DNA-binding domain in ENVOY its effect is likely to be executed via protein-protein interaction with one or more transcription factors. Also, it seems possible that the response to light is not exclusively dependent on one specific transcription factor and that the modulating function seen for many genes of this study might be performed via factors at a different level in the signal transduction cascade.

### Stimulation of growth on various carbon sources by light and ENVOY

**Figure 5:**
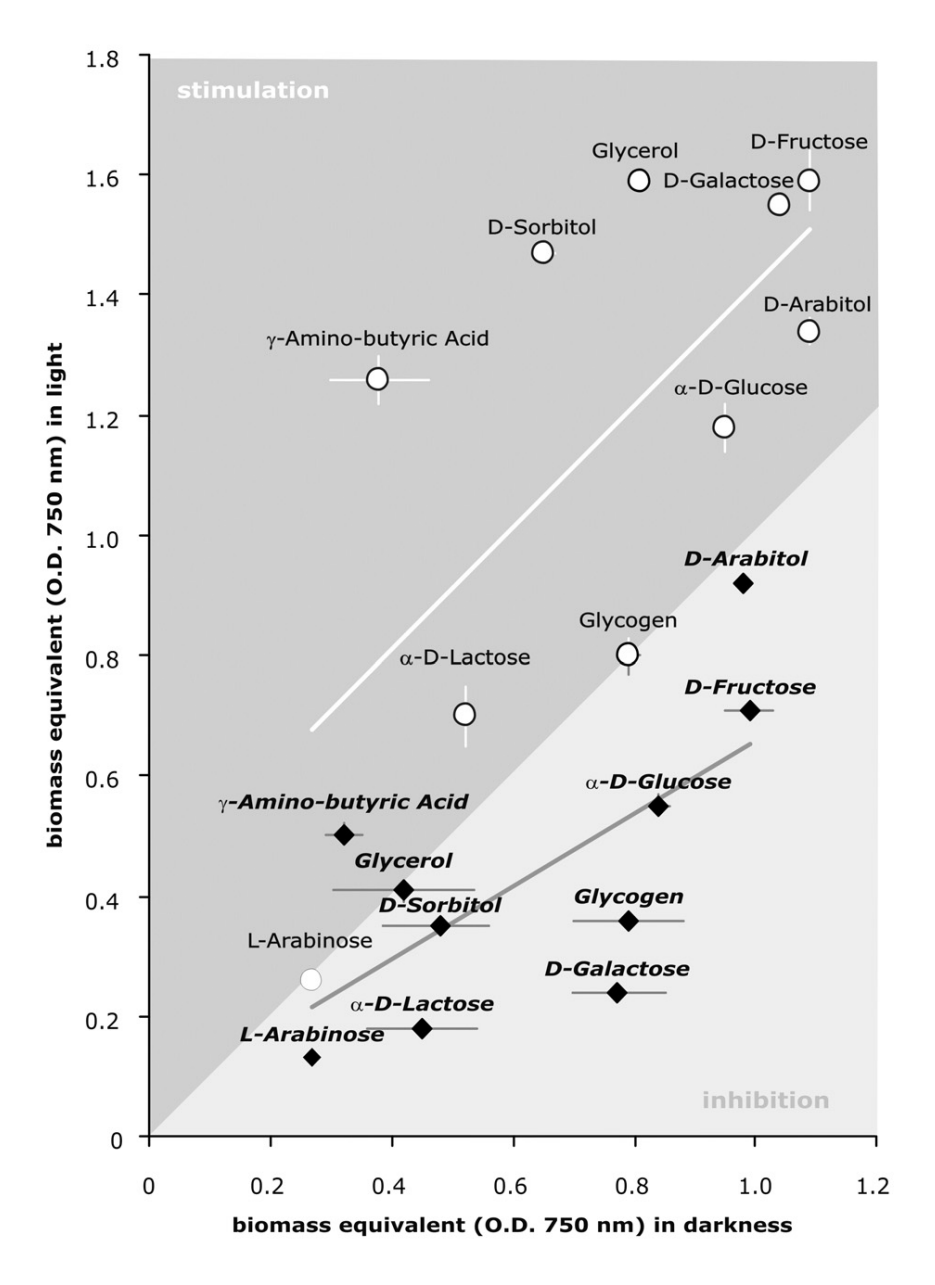
**Light stimulation of growth of *H. jecorina *on selected carbon sources, and the impact of ENVOY**. Biomass formation of wild-type strain QM9414 (open circles) or *env1*^PAS- ^(full diamonds) has been analyzed by the BIOLOG microplate assay. Biomass equivalents (OD_750_) after 72 hrs of growth are given. This time was chosen because then both strains were still in the phase of active growth on all carbon sources tested. The y-axes shows values obtained under constant light of 1800 lux, whereas the x-axes shows those obtained in constant darkness. Consequently, carbon sources on which no difference between growth in light or darkness occurs lie on the border between the shaded area (indicating light stimulation) and the open area (light inhibition). The two lines indicate the mean values for all carbon sources. All experiments were done in triplicates, standard deviation is indicated by bars.

Because of the up-regulation of energy metabolism by light in dependency of *env1*, we wondered whether this behaviour would also be reflected in an enhanced growth rate. We have therefore measured the growth rates of *H. jecorina *QM 9414 and the mutant strain *env1*^PAS- ^in light and in the dark on those carbon sources which enable highest growth rates by the parent strain [40]. The data, presented in Fig. 5, show that *H. jecorina *indeed grows faster on many of them in the presence of light, although to a variable degree. This enhanced growth rate was dependent on ENVOY, since no such stimulation was observed in the mutant strain *env1*^PAS-^. In fact, with the exception of growth on γ-aminobutyric acid, its growth rate was always lower in light than in the dark. When the data are compared between the two strains only in light and only in darkness (= i.e. the relative changes on the x- and y-axes are considered), it is evident that the two strains differ significantly stronger along the y-axes, thus indicating that light inhibits growth of strain *env1*^PAS-^. On the other hand, growth in darkness (with the exception of glycerol) was only very little affected (= both strains occur at similar positions at the x-axes). In the case of utilization of glycogen, whose position in the graph indicates light inhibition in the mutant strain, the results are in perfect agreement with the expression of *gph1 *(encoding a glycogen phosphorylase; see above Figure 1A): in the wild type strain, only a slight decrease in transcript abundance is observed in light as compared to darkness, but a more strongly decreased mRNA level is observed in the mutant strain *env1*^PAS- ^in light. These data are indicative of an *env1*-dependent enhancement of energy metabolism and thus biomass formation by light, and a negative effect of light on *H. jecorina *in the absence of functional ENVOY. It is thereby intriguing to note that this inhibition by light in the mutant strain *env1*^PAS- ^was not observed on all carbon sources (e.g. growth rates were similar on D-arabitol and glycerol, and on γ-aminobutyrate growth was even stimulated by light). The inhibitory effect of light in the absence of ENVOY is therefore carbon source dependent.

### Up-regulation of *env1 *is not sufficient for regulation of its target genes in darkness

**Figure 6:**
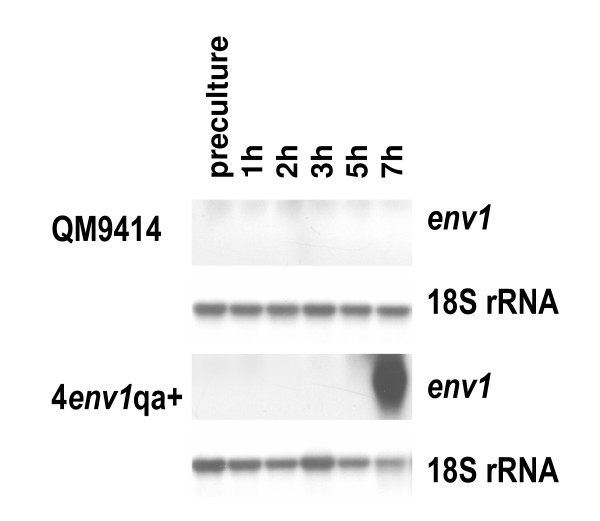
**Northern analysis of ENVOY overexpressing strains**. Strains were grown on Mandels Andreotti minimal medium with 1% (w/v) glycerol as carbon source in constant darkness and transcription of *env1 *in 4env1qa+ was induced by adding quinic acid to a final concentration of 0.6%. The parent strain QM9414 was used as control and treated equally. Mycelia were harvested after 1, 2, 3, 5 and 7 hours in darkness.

In order to find out whether up-regulation of *env1 *would be sufficient to induce transcription of its target genes in darkness, we introduced the *env1 *open reading frame under the control of the inducible *N. crassa qa2*-promoter into the *H. jecorina *parent strain QM9414, resulting in strain 4env1qa+. Strains were grown in constant darkness and expression of *env1 *was induced by addition of quinic acid to the parent strain and the *env1*-overexpressing strain. After quinate addition, induction of *env1 *transcription could be demonstrated (Figure 6). To elucidate whether this upregulation is sufficient for the expression of light induced, *env1 *dependent genes, we chose the *phr1 *gene encoding photolyase 1 as a model. Despite the strong transcription of *env1 *in the ENV1 overexpressing strains, no transcript indicating up-regulation of *phr1 *in darkness could be detected in these strains after addition of quinic acid (data not shown). Thus, despite the effect of the lack of functional ENVOY on transcription of *phr1*, overexpression of ENVOY is not sufficient for upregulation of *phr1 *in the darkness.

As a second model case, we wanted to test whether expression of *env1 *would be sufficient for the up-regulation of tre39031 in darkness and if the effect of the mutation of *env1 *in *env1*^PAS- ^(i.e. detectable transcript in darkness and decreased transcript levels in light; figure 1A) could be reversed by overexpression of *env1 *in constant darkness. Also in this case no transcript of tre39031 was detected in darkness and after induction of *env1*-transcription by quinic acid (data not shown). These results are in agreement with the assumption that ENV1 does not directly regulate the transcription of its target genes (at least not generally), but executes its function indirectly by interaction with transcriptional regulators, which are not available or inactive in darkness.

## Discussion

In this study, we used RaSH to isolate early light-responding genes from *H. jecorina*, which led to the identification of a total of 20 genes which were upregulated and 4 which were downregulated shortly after illumination. In addition, several of these genes were differentially effected by a mutation in the light regulatory protein ENVOY [22], whose closest neighbour *N. crassa *VIVID, is known to be a photoreceptor [17]. Besides ENVOY, VIVID is the only characterized PAS-domain protein of this type in fungi to date. To put the data obtained in this study in a genomic perspective: Rosales-Saveedra *et al.*[41] have recently used microarrays containing approximately one fifth of the genome of *Hypocrea atroviridis *(anamorph *Trichoderma atroviride*) for the screening of genes regulated by light, and identified 30 genes to be upregulated. This corresponds to 2.8% of the genes contained in the array used, and compares well to a value of 3% obtained for a similar study in *N. crassa *[8]. Only two of the genes which were found to be upregulated by blue light in *H. atroviridis *[41] were also upregulated in *N. crassa *[8], which may be explained by a significant difference in the physiology of these two fungi and the long phylogenetic distance between *Neurospora *and *Hypocrea/Trichoderma *spp. [42]. It is therefore interesting to note that only one of the genes (photolyase *phr1*) identified in this study was also found by Rosales-Saveedra et al. [41], although *H. jecorina *and *H. atroviridis *belong to the same fungal genus. The authors mentioned unpublished data that "several of the genes identified by them were also light upregulated in *H. jecorina*." This difference may be due to the missing of 80% of the genome in this study. On the contrary, the method of subtraction hybridization used in our study includes the whole transcriptome of the respective conditions to be compared, but from our experience also yields only a subset of all genes regulated under the conditions of interest. Hence we consider the present study complementary to that of Rosales Saveedra *et al.*[41]. Yet another explanation for the difference in the set of genes which were found to respond to light in these two fungi could be the fact that the ENVOY-homologue of *H. atroviridis *may not be functional: this assumption is supported by the following findings: first, its N-terminus is truncated at amino acids 1–6; second, it contains two upstream open reading frames close to the ATG (M. Schmoll, unpublished), which can profoundly influence the translation of the main ORF [43]. Finally, we could not detect the transcript of *H. atroviridis env1 *under several conditions where *env1 *is strongly transcribed in *H. jecorina *(data not shown). Taken together, this could reflect a different light regulatory machinery in these two closely related fungi.

Genes, which were actually upregulated by light and which required ENVOY for this process to function properly were the largest sample detected in this study (11 genes). One of them was the photolyase gene *phr1*, which has also been isolated from *H. atroviridis*, and which plays a role in the protection of genes against UV-light by photoreactivation of cyclobutan dimers of the pyrimidine nucleotides [44]. Rosales-Saveedra et al. [41] also reported the identification of a gene (*blu3*), which encodes a protein with an endonuclease III-type domain and which could function in excision repair. This gene was not identified in this study. However, another gene identified in this study (tre22454) encodes an ITP triphosphatepyrophosphatase, an enzyme responsible for the degradation of IMP and XMP, which accumulate as a result of cellular degradation of nucleotides which were modified by oxidative stress [46]. These data indicate that the early light response of *H. jecorina *involves reactions both against UV-light as well as oxidative stress. This is supported by the upregulation of tre42719 (encoding IMP dehydrogenase, an enzyme involved in the biosynthesis of nucleotide phosphates). Yoshida et al. [47] have shown that exposure of *N. crassa *to light evokes an oxidative stress response, in which nucleoside diphosphate kinase 1 plays a essential role e.g. by associating with a G-protein &-subunit for transmission of the light signal [48]. Finally, an increased demand for protection of the cell against a major threat is also evident from the upregulation of genes encoding components of cellular protein turnover such as tre16112 (a prenyltransferase required for ubiquitin biosynthesis), tre39031 (encoding a dipeptidyl peptidase III), and *ubi4 *(encoding polyubiquitin). The upregulation of an MSF toxin efflux pump (tre10571) with homology to proteins providing tolerance against fungicides in *Botrytinia fuckeliana *(DHA14 like major facilitator protein, AAF64435, E-value 3E-130; [49,50]) and *Mycosphaerella graminicola *(Mfs1, ABG57045, E-value 5E-127; [51]) upon illumination raises an intriguing question: is this defense-mechanism predominantly active in the presence of light i. e. during the day? If so, the efficiency of fungicides could be increased by carefully timing their application. However, we do not yet know whether the light regulation of this efflux pump also occurs in plant pathogenic fungi. In agreement with *H. atroviridis *[41], several of the genes which are upregulated by light encode genes involved in energy metabolism (tre9347, NAD synthase; tre20863, succinate dehydrogenase; tre39397, glucose transport), and regulation of all of them was influenced by *env1*. This is reminiscent of the findings by Kolarova *et al.*[52] that exposure of *T. viride *to light leads to increments in ATP levels and respiratory activity. Although this increased energy production could be required for the onset of photoconidiation upon exposition to light, this explanation is rather not applicable to *H. jecorina*, because this fungus does not need illumination for the induction of formation of conidia, and conidiates well in darkness. The detection of several genes involved in protein turnover to be responsive to light rather suggests that this enhanced energy demand reflects the physiological change in gene expression which is needed to adapt to light.

One light-responsive but not *env1*-dependent gene shows intriguing characteristics – tre45629: although the primary structure of the encoded protein is only poorly conserved, it contains all the signature sequences of a peptidyl arginine deiminase, an enzyme which converts arginine residues in proteins to citrulline, thereby altering the positive charge and hence the proteins ability to interact with other proteins and membranes. To the best of our knowledge, the role of this deimination has not yet been investigated in fungi. Moscarello *et al.*[53] have recently proposed that citrullinylation of myeline basic protein from brain is an important event in the pathogenesis of multiple sclerosis.

Another interesting finding was the detection that the cross pathway control protein CPC2 is regulated by light. Cross pathway control (CPC) of amino acid biosynthetic pathways is activated during amino acid starvation and also controls sexual development in *A. nidulans*. This activation is executed by the transcription factor CPC1/CPCA. In the presence of amino acids the pathway is repressed by the transcription factor CPC2/CPCB [54]. We therefore tested whether *H. jecorina cpc1 *would also respond to light. Interestingly, *cpc1 *did not respond to light, but is influenced by the presence of ENVOY. Thus, in *Hypocrea jecorina *the repressing factor is regulated by light but the activating factor is modulated by ENVOY. The coregulation of *cpc1 *and *hac1 *by ENVOY is also interesting in the context that Gcn4p (the *S. cerevisiae *orthologue of CPC1) is involved in the unfolded protein response [55], and that CPC1 was found to be upregulated during UPR in *H. jecorina *[56]. This suggests that ENVOY may be involved in the control of UPR.

Envoy – and particularly its putative counterpart in *N. crassa*, VIVID – have been described as proteins modulating the cellular response to light. However, we have shown here that this is only one of several roles which ENVOY apparently plays. Only eleven of the nineteen genes upregulated by light needed the function of *env1 *for this process. Five other genes showed an upregulation by light independently of *env1*, and in three genes the light-dependent upregulation was even stimulated in an *env1*-negative background. The nature of the proteins encoded by these genes did not yet provide us with an explanation for the specific role of these *env1*-independent and *env1*-repressed upregulations.

The results of this study point at an involvement of Envoy in the regulation of various cellular processes. Although based on this study we cannot differentiate between direct and indirect influences of ENVOY, it is obvious that this protein plays an important regulatory role at a central junction of signaling pathways. The finding that ENVOY is at least in some cases – as exemplified by the influence on *phr1 *and tre39031 – not able to execute its light-dependent function in darkness suggests that the presence of its putative interaction partners is required for a proper function of this regulatory mechanism. Similarly, also for *N. crassa *White collar-1 (WC-1) Lewis *et al.*[8] showed, that increased levels of WC-1 in darkness are not sufficient to activate all aspects of the phototransduction pathway. Since ENVOY comprises no known DNA-binding domain, it likely does not directly bind to DNA, and therefore executes its function via interaction with downstream regulatory proteins targeting the respective pathways. Thereby it could interact with either positive as well as negative regulatory factors, which would explain its positive and negative influences as shown in this paper. This interaction could also be influenced by the phosphorylation state of the casein kinase II phosphorylation sites in ENVOY (M. Schmoll, unpublished), and/or binding of the ligands to the PAS-domain of ENVOY. Since PAS domains are well known to be able to bind different ligands [57], ENVOY could thus execute its regulatory function both at a qualitative (conformational change due to bound ligand) and quantitative (expression efficiency) level. While the well characterized photoreceptors BLR1 and BLR2 (putatively as BLR1–BLR2 photoreceptor-complex) are predicted to mediate the reception of the light signal, this study reveals that ENVOY is involved in the conditional adaptation to light, because lack of functional ENVOY does not result in blindness but leads to an altered gene expression pattern of light-regulated genes. Such a function would well correspond with the finding of a gating function for the *N. crassa *orthologue VIVID [15]. In other words, Envoy most likely determines the significance of the light signal for a given cellular process under the current environmental conditions.

## Conclusion

The different responses to light, as demonstrated in this study, stress that light plays a role in several cellular processes of fungi, thereby displaying both positive and negative effects. Our data also emphasize that ENVOY has an apparently more widespread cellular role in this process than only in modulating the response to light. The importance of such a coordinator becomes apparent when it is considered that sunlight causes subsequent changes such as a rise in temperatures, decrease in humidity, and increase in UV light intensity. The adaptation to these environmental cues is of crucial importance in the evolution of every organism.

## Methods

### Microbial strains and culture conditions

The *H. jecorina *(*T. reesei*) wild-type strain QM9414 (ATCC 26921) and the *env1 *recombinant mutant lacking the PAS-domain (*env1*^PAS-^[22]) were used throughout this study. *H. jecorina *was grown in liquid culture in 1-L Erlenmeyer flasks on a rotary shaker (200 rpm) at 28°C in 200 ml of medium as described by [58] with 1% (w/v) glycerol as sole carbon source using 10^8 ^conidia/L (final concentration) as inoculum in constant darkness and harvested with red safety light or after the time of illumination (1800 lux; 25 μmol photons m^-2 ^s^-1^) as indicated with the figures. Cultivations of wild-type and mutant strain were done in parallel to ensure equal conditions.

*E. coli *JM109 [59] was used for the propagation of vector molecules and DNA manipulations.

### Preparation of PCR-Based cDNA Libraries

The experiment was performed essentially as described by Schmoll *et al.*[60] according to the RaSH method as published by [27]. For the driver cDNA mycelia were grown in constant darkness on minimal medium as described above for 24 hours, tester cDNA was prepared from mycelia exposed to light for 15 and 30 minutes and pooled.

### Reverse Northern Hybridization

For the Reverse Northern Hybridization, PCR products were loaded onto duplicate agarose gels and blotted with 0.4 N NaOH onto Hybond N membranes (Pall, New York, USA). Hybridization was performed using 2.5 μg of PCR amplified and subsequently radioactively labeled cDNA from tester or driver, respectively, as probes after *Eco*RII digestion and purification. The candidates for a more detailed analysis were chosen by visual inspection first, then this decision was cross-checked by quantitative measurements using the BIORAD Geldoc Imaging system (Bio-Rad, Hercules, California, US) and BIORAD Quantity One software, both for three different expositions of the blot.

### Nucleic acid isolation and hybridization

Fungal mycelia were harvested by filtration, washed with tap water, frozen and ground in liquid nitrogen. For extraction of DNA, mycelial powder was suspended in buffer A (1.2 M NaCl, 5 mM EDTA, 0.1 M Tris-HCl, pH 8.0), incubated for 20 min at 65°C, cooled down on ice, mixed with 1 vol. phenol:chloroform:isoamylalcohol 49:49:2 (v/v/v) and centrifuged (21000 g, 15 min). Following an extraction with 1 vol. of chloroform:isoamylalcohol 24:1 (v/v), the DNA was precipitated with 1 vol. of isopropanol and washed with 70% (v/v) ethanol. Total RNA was isolated by the guanidinium thiocyanate method [60,61]. Standard methods [62] were used for electrophoresis, blotting and hybridization of nucleic acids. The transcription pattern of *env1 *[22] under the respective conditions was used as a control hybridization for appropriate conditions with every cultivation. In case of unclear results or small signal differences, the hybridizations were repeated with samples from different, independent cultivations.

Normalization of gene expression was performed according to the following formula:

{(transcript abundance of gene x at time point)/(transcript abundance of gene x in wild-type in darkness)}/{(control 18SrRNA, time point)/(18S rRNA wild-type, darkness)}. The quantitative measurements were performed using the BIORAD Geldoc Imaging system and BIORAD Quantity One software from different expositions of the respective film. For every set of Northern blots one 18S rRNA hybridization was included as a loading and blotting control and used for quantification of the respective films.

### Sequence analysis and identification

The most promising candidates for light responsive genes showing clear differential transcription in the Reverse Northern Blot were selected for sequencing. PCR products as used for reverse Northern blotting were sequenced using primer RaSH1R, which binds within pBluescript immediately upstream of the inserts to be analyzed [60]. The respective sequences were used for BLASTX searches of the *T. reesei *genome database v2.0 [63]. For the genes identified thereby, protein sequences as provided by this database were used for a search for conserved domains in CDD [64,65] and for the nearest neighbour with NCBI Blastp [66,66,68]. If an E-value below 1E-30 for the Blastp result or 1E-10 for the result of the CDD search was obtained for a certain gene, the result was considered to assign a putative function. 1000 bp of the promoter sequence upstream of the first ATG of the respective open reading frame as predicted in the genome database were analyzed for known regulatory motifs.

### Biolog Phenotype Array analysis

Growth rates on selected carbon sources were investigated by means of the Biolog FF MicroPlate™ assay (Biolog Inc., Hayward, CA) as described by Druzhinina *et al.*[40]. Inoculated microplates were incubated in constant light (1800 lux, 25 μmol photons m^-2 ^s^-1^) or in the dark at 28°C, and percent absorbance at 750 nm determined in 12 h intervals between 36 and 72 h. Analyses were repeated at least three times for each strain.

### Construction of strains overexpressing *env1*

For inducible expression of ENV1 we introduced the open reading frame of *env1 *into the *Sma*I-site of the vector pmyx2 [69], resulting in *env1 *being under the control of the *N. crassa qa-2 *promoter which can be induced by addition of quinic acid to the culture medium to a final concentration of 0.6%. The resulting construct was transformed into the wild-type strain QM9414. Two positive strains were selected by PCR screening and Southern blotting, pregrown for 24 h on 1% (w/v) glycerol in darkness before adding quinic acid and transcript abundance was analyzed at several time points after addition of quinic acid in constant darkness. The wild-type strain was used as a control in parallel to those strains and was treated equally.

## Authors' contributions

AS performed Northern analyses and bioinformatic analysis of the genes described. CPK participated in the bioinformatic analysis and strategic planning of the work and drafted the manuscript. MAF carried out the BIOLOG-analysis and – together with ISD – performed the analysis of the respective data. MS performed Rapid Subtraction hybridization, Reverse Northern blotting, prepared the ENV1 overexpressing strains, participated in the data analysis and strategic planning of the work and wrote the final version of the manuscript.
